# Class 1 Sugar Beet Phytoglobin Shows Strong Affinity to Glyceraldehyde-3-Phosphate Dehydrogenase and DNA In Vitro

**DOI:** 10.3390/ijms26199404

**Published:** 2025-09-26

**Authors:** Leonard Groth, Miho Oda, Leif Bülow

**Affiliations:** Division of Pure and Applied Biochemistry, Lund University, 221 00 Lund, Sweden; leonard.groth@tbiokem.lth.se (L.G.); miho.oda.1530@tbiokem.lth.se (M.O.)

**Keywords:** phytoglobin, glyceraldehyde-3-phosphate dehydrogenase, nucleic acid, sugar beet, heme pocket, high ambiguity driven protein–protein docking, spectral shift analysis

## Abstract

Class 1 phytoglobins (Pgbs) are known for their multifunctional roles in plant stress responses, with recent studies suggesting broader interactions involving metabolic and transcriptional regulation. Interestingly, glyceraldehyde-3-phosphate dehydrogenase (GAPDH) moonlights in many roles in colocalized spaces during cellular stress that are strikingly suitable for supporting Pgb function. This study investigates the molecular interactions of class 1 Pgb from sugar beet (*Beta vulgaris*), BvPgb 1.2, and an alanine-substituted mutant (C86A), focusing on their ability to bind GAPDH and DNA. Using dual-emission isothermal spectral shift (SpS) analysis, we report strong binding interactions with GAPDH, with dissociation constants (K_D_) of 260 ± 50 nM for the recombinant wild-type protein (rWT) and a significantly stronger affinity for C86A (120 ± 40 nM), suggesting that the cysteine residue limits the interaction. Remarkably strong DNA-binding affinities were also observed for both variants, displaying biphasic binding. This behavior is characteristic of hexacoordinated globins and reflects the presence of two distinct species: a fast-reacting open pentacoordinated form and a slow-reacting closed hexacoordinated form with high apparent affinity. Here, the K_D_ in the open configuration was 120 ± 50 nm and 50 ± 20 nM for rWT and C86A, respectively. In the closed configuration, however, the cysteine appears to support the interaction, as the K_D_ was measured at 100 ± 10 pM and 230 ± 60 pM for rWT and C86A, respectively. Protein–protein docking studies reinforced these findings, revealing electrostatically driven interactions between BvPgb 1.2 and GAPDH, characterized by a substantial buried surface area indicative of a stable, biologically relevant complex. Protein–DNA docking similarly confirmed energetically favorable binding near the heme pocket without obstructing ligand accessibility. Together, these findings indicate a potential regulatory role for BvPgb 1.2 through its interaction with GAPDH and DNA.

## 1. Introduction

Phytoglobins (Pgbs), traditionally recognized for their roles during hypoxic stress through nitric oxide (NO) scavenging in the Pgb/NO cycle [1], have been reported to localize to nuclei and chloroplasts in *Lotus japonicus* and *Arabidopsis thaliana* [2]. Proteomic and metabolomic analysis of barley (*Hordeum vulgare*) seedlings overexpressing Pgb during hypoxia indicated that Pgbs improved survivability and prevented biomass/chlorophyll loss, as well as radical oxygen species (ROS) production [2,3]. Importantly, recent findings highlight specific interactions between Pgbs and histone proteins, particularly histone H2B, suggesting a direct regulatory mechanism involving chromatin remodeling. Under hypoxic conditions, class 1 Pgbs notably influence histone abundance, significantly elevating the levels of H2B histones. This regulatory effect appears distinct from classical acetylation or methylation modifications, hinting at novel pathways mediated by Pgbs [3].

The discovery that class 1 Pgbs from *L. japonicus* and *A. thaliana* can accumulate in chloroplasts despite lacking recognizable N-terminal transit peptides suggests the existence of an uncharacterized protein trafficking mechanism. Indeed, transient expression studies have revealed that closely related globins such as LjPgb3.1 and LjPgb3.2, sharing 83% sequence identity, differ markedly in their chloroplast localization, implying functional specificity and nuanced organelle recognition mechanisms beyond canonical peptide signaling [2].

Intriguingly, glyceraldehyde-3-phosphate dehydrogenase (GAPDH), historically considered a canonical metabolic enzyme, has emerged as a highly multifunctional moonlighter [4], involved in diverse processes including transcriptional regulation and heme trafficking [5]. Recently, GAPDH was shown to facilitate the maturation and transport of heme proteins such as hemoglobin (Hb) and myoglobin (Mb) [6]. This raises the possibility that GAPDH could similarly mediate heme insertion in Pgbs, which could be supported by determining the degree of affinity between the two proteins. Apart from roles in synthesis of heme proteins, GAPDH has been identified as a crucial component of OCA-S, a multicomponent transcriptional coactivator essential for the S-phase-specific transcription of histone H2B. This coactivator’s activity is uniquely modulated by the cellular redox state [7], further underscoring a direct functional link between metabolic status and histone transcription. Remarkably, GAPDH has also been shown to indirectly maintain NAD+ homeostasis in nuclei during stress by translocating nicotinamide phosphoribosyltransferase (NAMPT), which constitutes a part of the nicotinamide salvage pathway [8]. Like many class 1 Pgbs, NAMPT also lacks N-terminal transit peptides [9]. NADH is also, coincidentally, consumed during the Pgb/NO cycle [10].

This convergence of stress-induced roles for GAPDH—heme maturation of globins, nuclei-NAD+ homeostasis, protein transport, and H2B transcription—suggests a dynamic interplay between GAPDH and Pgb. Furthermore, GAPDH is known to adopt a range of different roles during stress conditions [11] that also robustly induce Pgb expression, such as nitrosative (via S-nitrosylation) and oxidative stresses [12,13,14]. Unlike animals, which have a single GAPDH isoform, plants encode multiple isoforms (*gapA*, *gapB*, *gapC*, and *gapCp*) localized in different compartments. The gapA and gapB products form the chloroplastic A_2_B_2_- and A_4_-GAPDH isozymes that function in the Calvin–Benson cycle [15].

In contrast to the well-established multiple roles of animal GAPDH, the multifunctionality of cytosolic GAPDH in plants (GAPC) remains less understood [11]. GAPC is a target of diverse redox modifications, which may regulate its subcellular localization. Increasing evidence suggests that, like its animal counterpart, GAPC carries out non-catalytic roles consistent with moonlighting behavior [11]. Given that globins such as BvPgb 1.2 are nuclear-encoded and cytosolically expressed [13], GAPC is more likely than the plastidic isoforms to function in a chaperone capacity.

GAPDH’s ability to shuttle between different subcellular compartments, coupled with its involvement in cellular redox sensing and stress response, positions it uniquely to interact with Pgbs under conditions of oxidative or nitrosative stress. GAPDH [16] and globins [17,18] have coexisted for billions of years, giving ample time for sophisticated and biologically relevant regulatory patterns to evolve.

Like GAPDH, globins are multifunctional agents. Hb and Mb, for example, exhibit DNA-binding capabilities and endonuclease-like activities, particularly when oxidized to their ferryl (Fe(IV)) forms [19,20]. Structural analyses have additionally revealed unexpected parallels between globins and DNA-binding domains of bacteriophage repressors, suggesting an evolutionarily conserved potential for nucleic acid interactions among globin-fold proteins [21].

In this study, we explore these multifunctional roles in the context of class 1 Pgbs from sugar beet (*Beta vulgaris*), namely the recombinant wild-type (rWT) of BvPgb 1.2 (PDB: 7ZOS) and its cysteine-to-alanine variant (C86A, PDB: 7Z1U). The C86A was included as prior research on the conserved cysteine residue has revealed functional roles in redox activities unrelated to structure [22,23]. This study employs BvPgb 1.2 and C86A as model proteins to assess whether this cysteine impacts binding affinities to GAPDH and DNA. This was performed due to the well-characterized protective profile of the cysteine residue in BvPgb1.2 [22,23,24]. We employed a microscale capillary-based isothermal spectral shift (SpS) assay to determine ligand binding. SpS detects ligand-induced blue- or red-shifts in fluorescence spectra under isothermal conditions using dual-wavelength detection at 650 and 670 nm. The fluorescence intensity ratio (670/650 nm) is plotted against ligand concentration to derive dissociation constants (K_D_). This method has been previously applied to protein–protein interactions [25,26]; here, we adapted it to quantify binding of BvPgb1.2 and its C86A variant to GAPDH and DNA under defined assay conditions. Through these SpS measurements, we demonstrate that BvPgb 1.2 can directly interact with both GAPDH and DNA, independent of the semi-conserved cysteine residue. These results were further supported by high-ambiguity-driven protein–protein docking. Our findings thus shed light on a potential complex subcellular trafficking and functional dynamics of Pgbs, advancing our understanding of their roles in plant cellular responses to oxidative stress.

## 2. Results

### 2.1. BvPgb 1.2 Complex Measurements Using Isothermal Spectral Shift Assays

BvPgb 1.2 and C86A were produced in recombinant *E. coli* and purified using an established two-step chromatographic strategy [23], yielding preparations of >95% purity. To enable subsequent analysis, the Pgbs were modified by covalent attachment of an N-hydroxysuccinimide (NHS)-ester-activated dye to accessible primary amines, including lysine side chains and the N-termini.

**Figure 1:**
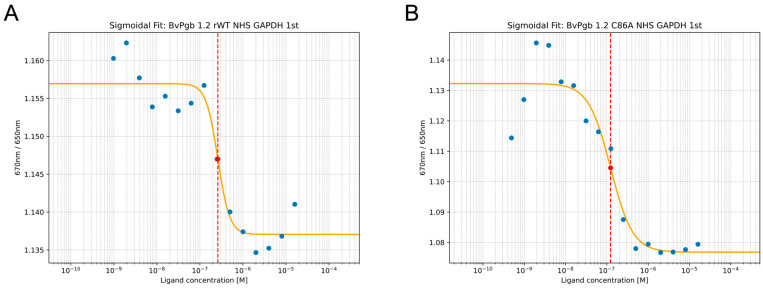
Glyceraldehyde-3-phosphate dehydrogenase (GAPDH) affinity to (**A**) recombinant wild-type (rWT) of class 1 phytoglobin from *Beta vulgaris* (BvPgb 1.2) and (**B**) its cysteine-to-alanine substituted variant (C86A) can be well characterized using isothermal spectral shift (SpS), with transitions and corresponding dissociation constant (K_D_, dotted red line) highlighted on clear dose–response curves (measurements: blue dots, sigmoidal fit: orange line).

First, we aimed to assess the biochemical interaction between BvPgb and GAPDH. Using SpS, we determined the K_D_ of the rWT Pgb-GAPDH complex to be 260 nM ± 50 nM. Interestingly, the K_D_ measured for the C86A mutant revealed a halved K_D_ at 120 ± 40 nM, suggesting that the cysteine residue limits binding (see Figure 1).

**Figure 2:**
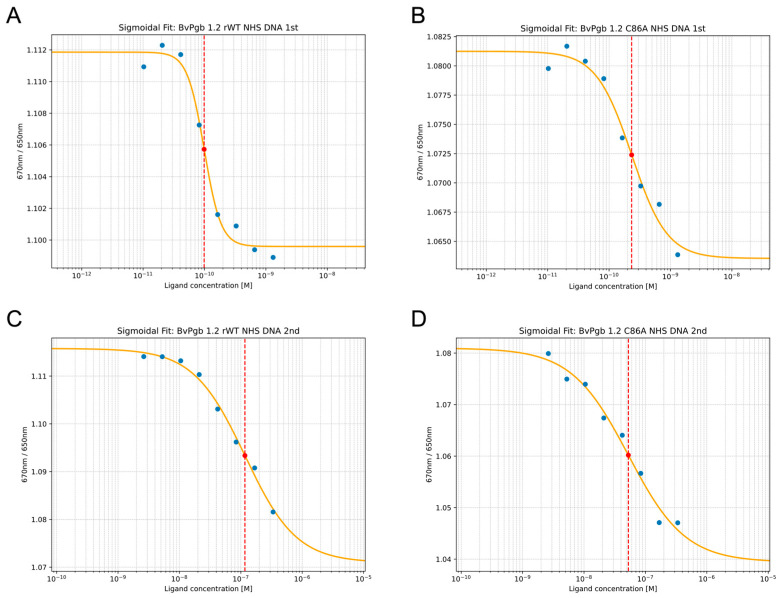
DNA affinity to BvPgb 1.2 rWT (**A**,**C**) and C86A (**B**,**D**) can be well characterized using isothermal SpS. Two transitions or dose–response curves were clearly observed for BvPgb 1.2 rWT, with transitions and corresponding K_D_s (red dotted line) for the rWT highlighted in (**A**,**C**) for the first and second transitions, respectively, and similarly for C86A in (**B**,**D**).

Next, we examined how well BvPgb 1.2 could bind DNA, and we performed similar measurements to determine the K_D_ for both rWT and the C86A mutant. In this study, we used plasmid DNA as target to obtain a well-defined nucleic acid target. In this case, BvPgb 1.2 revealed a similar biphasic behavior (see Figure 2) previously seen for other hexacoordinated globins [27,28,29]. Hexacoordinate globins are thought to interconvert between two conformational states: an open, metastable form in which the distal histidine readily dissociates, creating a reactive pentacoordinate heme capable of rapid ligand binding, and a closed, more stable form where conformational constraints limit ligand access and slow binding. The stable/closed and metastable/open reaction-scheme for hexacoordinate globins [27] likely holds here as well, since the heme center drives the endonuclease activity of globins [30].

The K_D_s of 100 ± 10 pM and 120 ± 50 nM for rWT, and 230 ± 60 pM and 50 ± 20 nM for the C86A suggest an unusually strong affinity for DNA.

The results from the SpS measurements of both the BvPgb 1.2–GAPDH complex and the BvPgb 1.2–DNA complex for both the rWT and C86A variants are compiled in Table 1 below. Notably, the cysteine residue appears to limit interaction with DNA only in the “open” configuration.

### 2.2. BvPgb 1.2–GAPDH Docking Analysis

To further investigate the observed interaction between BvPgb 1.2 and GAPDH, we performed protein–protein docking using the high-ambiguity-driven protein–protein docking platform HADDOCK 2.4. This yielded 378 structures distributed across 7 clusters, corresponding to 94% of the generated water-refined models.

**Figure 3:**
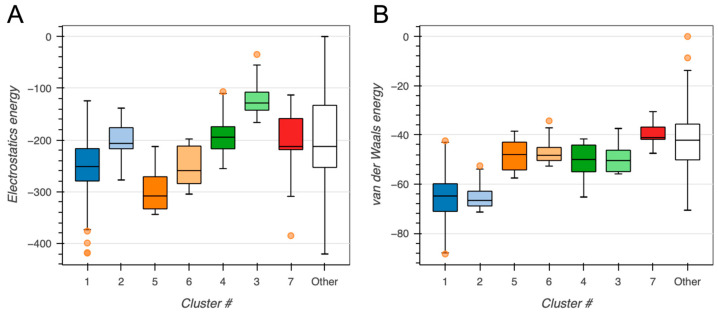
BvPgb 1.2–GAPDH docking clusters. Box plots show the distribution of (**A**) electrostatics energy and (**B**) van der Walls energy (Evdw) for each identified cluster. Outliers are shown as orange points. All energy terms are given in kcal/mole.

To assess the quality and convergence of the generated protein–protein docking models, we evaluated the distribution of key energetic terms across all predicted clusters. Electrostatic and van der Waals (vdW) interactions were first analyzed using box plots to identify clusters with favorable interaction profiles (see Figure 3).

**Figure 4:**
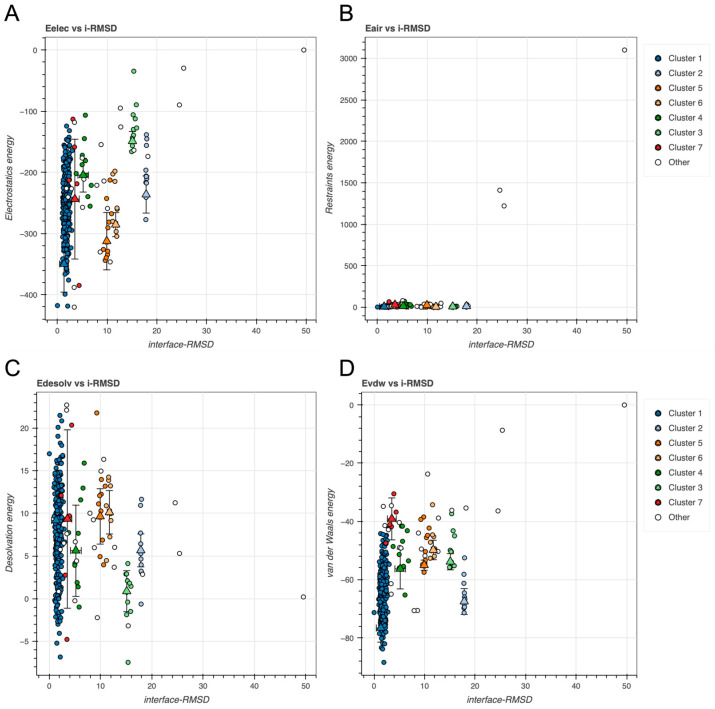
Scatterplots of energy components vs. interface root-mean-squared deviation (i-RMSD) (Å) for GAPDH docking models across identified clusters. (**A**) Electrostatics energy (Eelec), (**B**) restraints energy (Eair), (**C**) desolvation energy (Edesolv), and (**D**) Evdw. All energy terms are given in kcal/mole. Triangles and error bars represent cluster means and standard deviations.

Next, we examined the relationship between interface root-mean-square deviation (i-RMSD) and individual energy components to determine whether energetically favorable clusters also coincided with geometrically near-native poses. Scatterplots of electrostatics, restraint violations, desolvation, and vdW energies versus i-RMSD revealed clear energy-RMSD correlations for several clusters, particularly for electrostatics and vdW components (see Figure 4). Clusters with lower restraint energy and lower i-RMSD values suggest good satisfaction of the input ambiguous interaction restraints and consistent sampling.

Clusters with lower electrostatic energy tend to show lower i-RMSD, indicating more favorable and specific charge complementarity at the interface (see Figure 4A). Clusters with both low Eair and low i-RMSD indicate models that are geometrically close and restraint-consistent (see Figure 4B). Lower desolvation energies correspond to better packed interfaces (see Figure 4C), whereas vdW highlights clusters with compact sterically favorable interactions (see Figure 4D).

**Figure 5:**
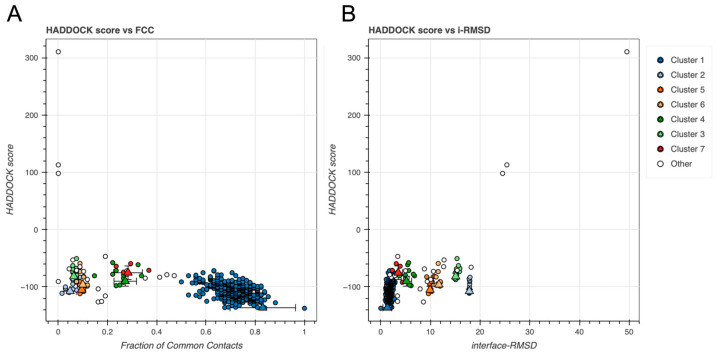
HADDOCK score comparisons for GAPDH docking models across clusters against (**A**) fraction of common contacts (FCC) and (**B**) i-RMSD (Å). Triangles and error bars represent cluster means and standard deviations.

Finally, to evaluate overall docking performance, we examined the HADDOCK score in relation to both fraction of common contacts (FCC) and i-RMSD (see Figure 5). HADDOCK score is a weighted sum of electrostatics energy (Eelec), restraints energy (Eair), desolvation energy (Edesolv), and vdW energy (Evdw) [31]. FCC reflects how similar the docking models are to each other within a cluster (see Figure 5A), whereas i-RMSD reflects how closely the docked interface aligns with the native interface geometry (see Figure 5B). These global comparisons allowed identification of clusters that were energetically favorable and structurally consistent [31,32,33,34].

Cluster 1 displayed best performance (see Figure 3, Figure 4 and Figure 5), composing 324 of the 378 structures (~86%), obtaining a highly favorable HADDOCK score of −136.7 ± 0.6, and a Z-score of −2.0. Structural convergence within the cluster was high, as reflected by the low RMSD from the overall lowest-energy structure (1.4 ± 0.8 Å).

Energetic analysis revealed strong electrostatic interactions (–349.9 ± 39.9 kcal/mole) and substantial vdW contributions (–76.6 ± 4.2 kcal/mole) offset by a modest desolvation penalty (+9.2 ± 4.9 kcal/mole). Importantly, restraint violations were minimal (7.0 ± 3.0 kcal/mol), suggesting good structural compatibility and compliance with input definitions of the putative interaction interface.

The average buried surface area (BSA) for cluster 1 was 2463.4 ± 146.5 Å^2^, more than 25% of the surface of BvPgb 1.2 monomer.

Relevant statistics from other clusters with negative Z-score are compiled in Table 2 below.

**Figure 6:**
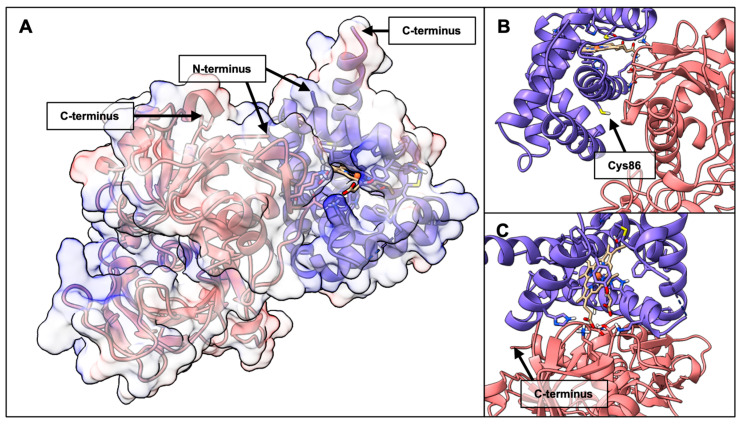
Visualization of the docked complex between BvPgb 1.2 rWT (blue, PDB ID: 7ZOS) and GAPDH (red, PDB ID: 1U8F, O-chain). (**A**) Full view of the complex in electrostatic surface representation with 50% transparency on the relevant chains of the GAPDH-Pgb complex, with N- and C-termina indicated by arrows. The heme group is displayed in atomic stick representation. (**B**) View of cysteine residue, indicated by an arrow, in relationship to GAPDH. (**C**) Front view of the heme pocket. It binds to the Pgb such that the heme pocket remains accessible to solvent and potential ligands with the C-terminus of GAPDH indicated.

Structural inspection of the top candidate within Cluster 1 revealed that GAPDH binds near the heme pocket of BvPgb 1.2, in such a way that the heme pocket remains exposed to solvent (see Figure 6).

### 2.3. BvPgb 1.2–DNA Docking

**Figure 7:**
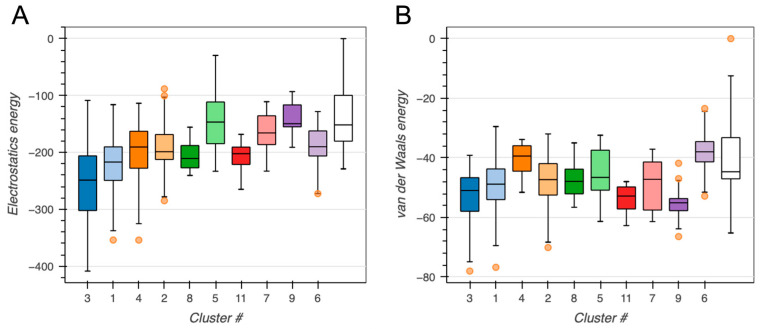
BvPgb 1.2–DNA docking clusters. Box plots show the distribution of (**A**) electrostatics energy and (**B**) vdW energy for each identified cluster. Outliers are shown as orange points.

To further investigate the BvPgb 1.2 and DNA (PDB ID: 1BNA) interactions, we performed protein–DNA docking. This yielded 358 structures in 11 clusters, representing 89% of the generated water-refined models. To assess these models, we performed the same evaluation as in the protein–protein dock (see Figure 7). This revealed a higher degree of homogeneity across clusters, compared to the GAPDH dock.

**Figure 8:**
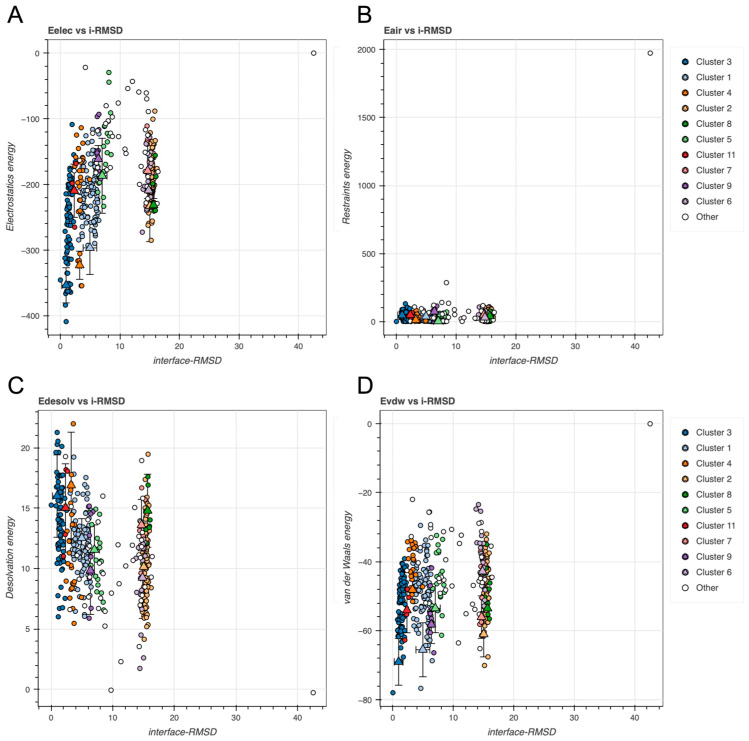
Scatterplots of energy components vs. i-RMSD (Å) for DNA docking models across identified clusters. (**A**) Eelec, (**B**) Eair, (**C**) Edesolv, and (**D**) Evdw. Triangles and error bars represent cluster means and standard deviations. All energy terms are given in kcal/mole.

Like GAPDH, the relationship between i-RMSD and individual energy components revealed several energetically favorable clusters (see Figure 8).

**Figure 9:**
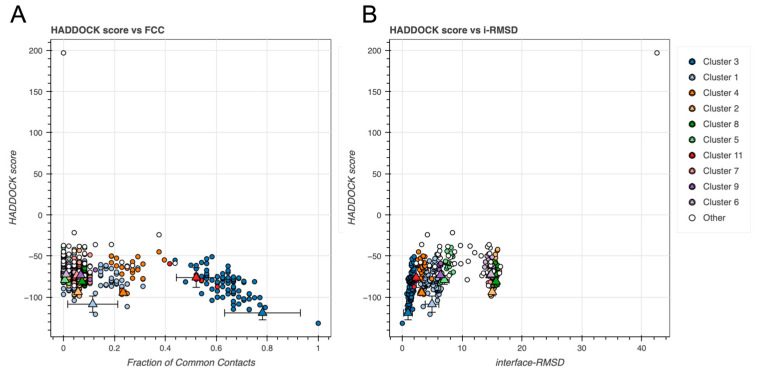
HADDOCK score comparisons for DNA docking models across clusters against (**A**) FCC and (**B**) i-RMSD (Å). Triangles and error bars represent cluster means and standard deviations.

The overall docking performance was likewise evaluated by looking at the HADDOCK score against the FCC and i-RMSD (see Figure 9).

Cluster 3 displayed best performance (see Figure 7, Figure 8 and Figure 9), composing 80 of the 358 structures (~21%), with a favorable HADDOCK score of −119.1 ± 7.3, and a Z-score of −2.1.

Cluster structural convergence was high, with the overall lowest-energy structure at 0.9 ± 0.6 kcal/mole. Modest desolvation penalty (16.0 ± 3.0 kcal/mole) and restraint violations (46.9 ± 26.5 kcal/mole) are offset by electrostatic (−353.5 ± 23.2 kcal/mole) and vdW (−69.0 ± 5.9 kcal/mole) energy contributions, like the GAPDH dock, suggesting good structural compatibility.

The BSA for Cluster 3 was 1701.4 ± 37.9 Å^2^, a substantial amount of the surface of the BvPgb 1.2 monomer. The statistics for clusters with negative Z-value are tabulated below (see Table 3). Together, they account for 284 of the 358 (~79%) water-refined structures.

**Figure 10:**
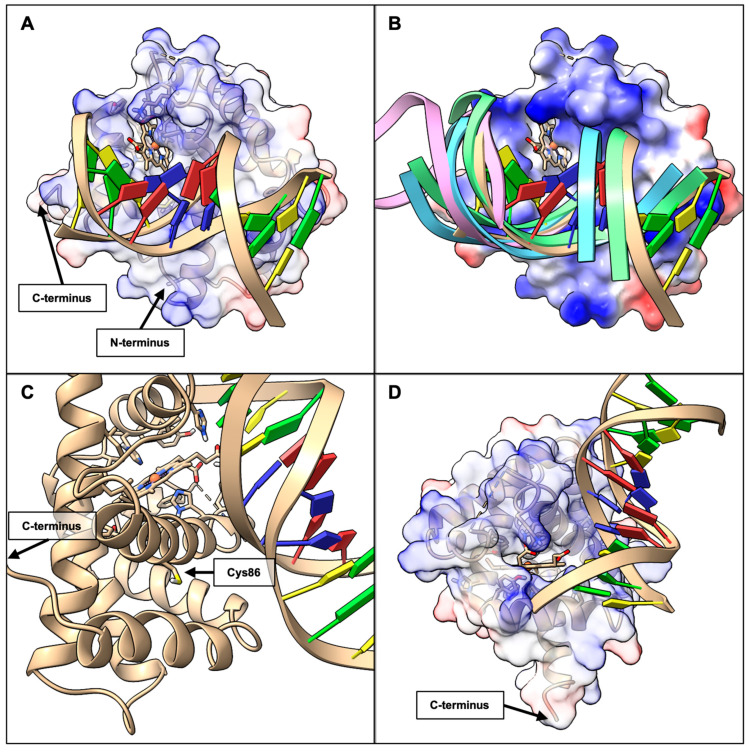
Visualization of the docked complex between BvPgb 1.2 rWT and DNA (PDB ID: 1BNA). Heme group displayed in atomic stick representation, nucleic acids represented as slabs, and protein represented as cartoon ribbons. (**A**) Full view of a representative structure from Cluster 3 (tan), with the protein surface represented as a 50% transparent electrostatic surface, with a major groove oriented directly in front of the open heme cavity, with N- and C-termina indicated by arrows. (**B**) View of the representative structure of Cluster 3 (tan) and backbone of nucleic acids from Cluster 1 (blue), 2 (pink), and 4 (green), with protein represented as an opaque electrostatic surface. (**C**) View of C86, as indicated by an arrow, in relationship to DNA. (**D**) A different angle of the representative structure from Cluster 3, with the C-terminus indicated by an arrow and display settings similar to (**A**).

Structural inspection of the docked models revealed DNA binds near the heme pocket of BvPgb 1.2 (see Figure 10), leaving the heme moiety accessible like in the GAPDH case (see Figure 6). This is true for all clusters with negative Z-value, where the DNA traverses and rotates accordingly along the Pgb interface (see Figure 10B).

### 2.4. Structural Similarity of Predicted GAPC to Human GAPDH

**Figure 11:**
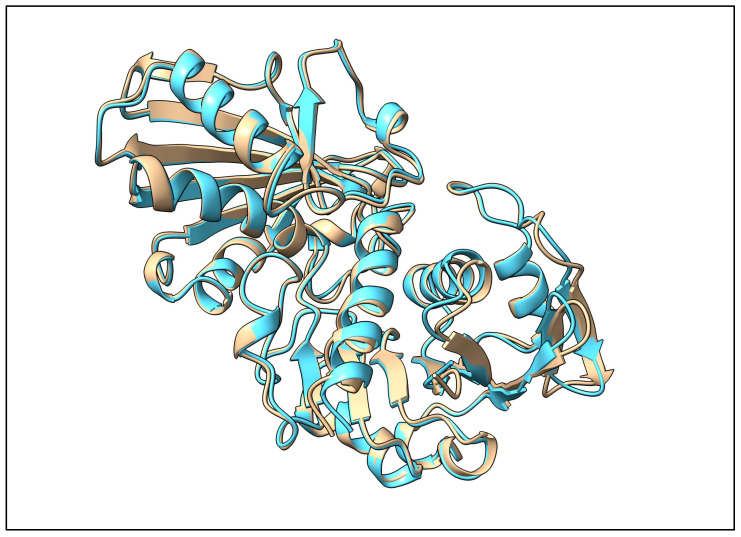
Predicted structure of putative *B. vulgaris* GAPC (tan) aligned to O-chain of GAPDH (PDB: 1U8F) (blue).

The *A. thaliana* GAPC (UniProt accession P25858) [35] shares 92.5% sequence identity with a hypothetical protein (transcript ID: KMT11247) annotated in the *B. vulgaris* RefBeet-1.2.2 genome assembly [36]. The AlphaFold3-predicted structure [37] of this putative *B. vulgaris* GAPC aligns very well with the O-chain of GAPDH used in our docking analysis (see Figure 11). The alignment through UCSF ChimeraX matchmaker [38] yielded an RMSD of 0.49 Å over 324 pruned Cα pairs (0.73 Å over all 333 pairs).

## 3. Discussion

The present study provides evidence for previously unexplored molecular interactions of BvPgb 1.2. We have made structural models to explain binding affinities between BvPgb 1.2 and GAPDH/DNA measured by SpS. Human GAPDH was used in the experiments, and docking was similarly performed on this enzyme. The very high structural similarity between the putative *B. vulgaris* GAPC and GAPDH (see Figure 11) suggests that the used protein is interchangeable with the in planta GAPC, lending further merit to our experimental results. Previous studies on C86A make a strong case for the cysteine residue being relatively uninvolved with the structure of BvPgb 1.2 [23], instead modulating function through redox activity [22].

Both protein–protein and protein–DNA complexes were dominated by electrostatic and vdW interactions (see Figure 3 and Figure 7), indicating high sensitivity to salt and pH. Although measurements of cytosolic pH in plants are sparse, a recent whole-plant study in potato reported values of ~7.09 ± 0.14 in cortical cells and the root absorption zone [39]. This suggests that the pH of the used buffer is comparable to the cytosolic environment in planta. However, growing evidence also highlights pH as an important signal and secondary messenger in plants [40]. Given that BvPgb1.2 is induced by oxidative stress [13], pH is likely a relevant factor to consider, warranting further investigation.

Our SpS measurements identified distinct K_D_s for GAPDH interactions with both rWT (260 ± 50 nM) and the C86A mutant (120 ± 40 nM) of BvPgb 1.2. Both exhibited moderate affinity within the typical range for biologically relevant transient interactions [41]. For example, the K_D_ value for the GAPDH–lactoferrin interaction was determined to be 43.8 nM [42]. Intriguingly, the C86A mutant showed significantly enhanced affinity, suggesting that the cysteine residue at position 86 may impose constraints on GAPDH binding, in addition to its redox-sensitive regulatory mechanisms [22,23].

Benchmarked HADDOCK complexes, such as the glucose-specific enzyme IIA (E2A) in complex with the histidine-containing phosphocarrier protein (HPr), report scores comparable to those of Cluster 1 (see Table 2). E2A–HPr docked in free protein forms yielded intermolecular energies of –207 kcal/mole and a buried surface area (BSA) of 1434 Å^2^ [31].

In typical stable protein–protein complexes, a BSA greater than ~1500–2000 Å^2^ is considered indicative of specific, non-transient interactions [43,44]. The BSA observed here exceeds this threshold and, in combination with the strong electrostatic and vdW contributions, suggests that the Pgb–GAPDH interaction may have functional significance. Indeed, large interfaces exceeding 2000 Å^2^ tend to induce conformational changes in both species [43]. Our structural analysis revealed that GAPDH (see Figure 6) and DNA (see Figure 10) binding does not necessarily hinder ligand access to the heme pocket, suggesting that these complexes could form without fully compromising the functionality of Pgbs.

The DNA-binding properties of BvPgb 1.2 introduced an additional layer of functional diversity. Our results clearly demonstrate a biphasic binding behavior characteristic of hexacoordinated globins [14,27]. Another approach in determining affinities is through isothermal titration calorimetry (ITC). However, the relatively high sample requirement of ITC poses challenges when working with biological samples and hard-to-produce proteins [45]. These concerns are circumvented through SpS measurements, which have previously been used to determine binding affinities in BvPgb 1.2 and C86A [24].

Given their small size at ~19 kDa [23], monomeric and dimeric BvPgb 1.2 can enter nuclei by passive diffusion through nuclear pores without requiring a transit motif or nuclear localization signal [46]. Nuclear accumulation can thus be a result of the high affinity for DNA observed here, with closed-configuration BvPgb 1.2 having K_D_s in the pM range.

**Figure 12:**
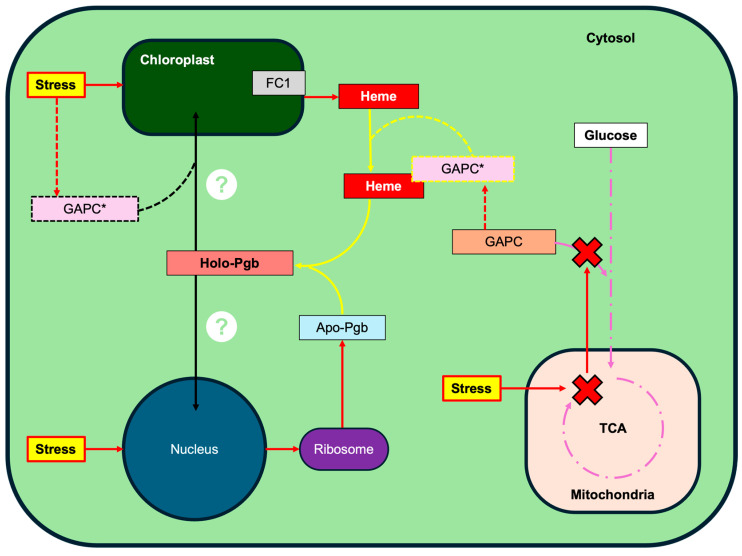
Schematic representation of proposed interactions. Solid lines represent well-established processes, and dotted lines represent proposed processes (see text). Stress (yellow box, red arrows) induces apo-Pgb (light blue box) expression, promotes extra-plastidic export of heme (stark red box) by ferrochelatase 1 (FC1, gray box), and prevents GAPC (orange box) from typical metabolic function (purple lines). Heme maturation (yellow arrows) of apo-Pgb to holo-Pgb (light red box) is herein suggested to be facilitated by moonlighting GAPC (GAPC*, pink box). The exact mechanism behind holo-Pgb trafficking is unresolved (question marks). This paper suggests that Pgb transport (black lines) to nucleus can be explained by inherent affinity to DNA, whilst intra-plastidic transport could be facilitated by GAPC* activity, as elaborated in text.

How Pgbs are trafficked to chloroplasts, however, remains elusive [2]. Unlike the predominantly mitochondrial C4-pathway for heme synthesis used by animals, plants make heme within the chloroplast through the C5-pathway [47,48]. This pathway is shared with chlorophyll synthesis and diverges from that route at the incorporation of ferrous Fe(II) by ferrochelatase (FC) [49], of which plants make two isoforms, FC1 and FC2. Expression analysis reveals that whilst FC1 is expressed universally, FC2 appears only in photosynthetic tissue [50,51]. Since FC1 is localized to the envelope membrane of chloroplasts and also co-induced with cytosolic heme proteins, it is presumed to be responsible for supplying cytosolic heme [52,53]. In this capacity, FC1 is also thought to act in the retrograde signaling pathway underpinned by cytosolic heme. Although the precise extra-plastidic transport mechanism for heme remains unclear [54], the cytoplasmic fraction of heme likely contributes to stress defense and intracellular signaling during acclimation [54]. If GAPDH is responsible for heme maturation of Pgbs, as it is for Hb and Mb [6], it could plausibly play an additional chaperone-role for an uncharacterized plastidic trafficking route. A schematic representation of the potential interplay between stress, heme-transport, GAPC, and Pgb is presented in Figure 12 below. Further research is required to clarify this potential role.

Interestingly, the C86A mutation impacted DNA-binding affinity differently in the open (K_D_ of 120 ± 50/50 ± 20 nM for rWT/C86A, respectively) and closed configurations (K_D_ of 100 ± 10/230 ± 60 pM for rWT/C86A, respectively), increasing affinity in the open configuration while reducing it in the closed form. This indicates the cysteine residue’s dual role in both limiting and facilitating interactions depending on the structural context. It should be noted that the closed protein species can account for 70% of the total population in class 1 rice phytoglobins (rHb1) [27]. The large difference in affinity suggests that the functional relationship between DNA and Pgbs could potentially depend on the state of the Pgb in question. The impact pH and/or the cysteine residue has on the relative population of closed- vis-à-vis open-Pgbs has yet to be determined, providing an interesting avenue for future research.

Collectively, these findings broaden our understanding of Pgb functionality, positioning them as players in sophisticated cellular stress response networks. Future studies should aim to explore the precise cellular conditions under which these interactions occur and elucidate downstream biological implications, especially regarding the potential transcriptional regulatory roles of Pgbs in planta. Clearly, proteome complexity exceeds that of the genome, not only due to subcellular localization [55] but also due to conformational and subsequent functional changes due to environmental factors.

## 4. Materials and Methods

All water used had a resistivity of 18.2 MΩ·cm and total organic content of less than five parts per billion. All structural models were rendered in UCSF ChimeraX 1.10 [38]. All structures are available upon request.

### 4.1. Protein Production

*Escherichia coli* BL21(DE3) cells were transformed to express recombinant wild-type (rWT) and C86A mutant proteins and purified as described in previous work [24]. Protein purity was assessed by SDS-PAGE, where protein samples were mixed with 2× Laemmli sample buffer (final concentrations: 62.5 mM Tris-HCl, pH 6.8; 4% SDS; 20% glycerol; 5% β-mercaptoethanol; 0.02% bromophenol blue) and boiled at 95 °C for 5 min. Samples were resolved on 7.5% Mini-PROTEAN^®^ TGX™ Precast Protein Gels (15-well, 15 µL; Bio-Rad Laboratories, Hercules, CA, USA; Cat. No. 4561026) using the Mini-PROTEAN Electrophoresis Cell system (Bio-Rad Laboratories, Hercules, CA, USA) according to the manufacturer’s instructions.

### 4.2. Isothermal Spectral Shift Assay

Protein labeling and the isothermal SpS assay were performed as described previously [24]. Briefly, degassed phosphate-buffered saline (PBS) with a pH of 7.4 (cat# 21-040-CV; Corning, NY, USA) was supplemented with 0.05% Tween20. Globins were subject to buffer exchange, according to manufacturer instructions. A twofold molar excess of dye was added to the globins prepared to a final concentration of 20 µM. Prior to dying, both BvPgb 1.2 and GAPDH (cat# G6019; Sigma-Aldrich; St. Louis, MO, USA) were centrifuged at 20,000× *g* at 4 °C for 15 min. Labeled globins were kept in the dark and on ice awaiting capillary loading.

Sixteen-step dilutions of ligands (10 µL) were prepared in PCR-strip tubes, combined with equal volumes of labeled protein, and allowed to equilibrate for 15 min before being loaded onto premium capillaries (Cat# MO-K025; NanoTemper Technologies GmbH; München, Germany) and placed into the Monolith X (NanoTemper Technologies GmbH; München, Germany). DNA stock solution was prepared from purified pET19b vectors. The highest ligand solution concentrations were prepared at 673 nM for DNA (i.e., 2.5 ng_pET19b_/µL) and 32 µM for GAPDH. All SpS measurements in this study were performed at 25 °C using the Monolith X (NanoTemper Technologies GmbH; München, Germany) instrument equipped with dual-emission detection optics.

The fluorescence intensity ratio (670/650 nm) was plotted against ligand concentration to derive a K_D_ from a fit to the dose–response curve as described previously [24]. The standard deviation (σ_KD_) of log_10_(K_D_) was estimated as the square root of the corresponding diagonal entry of the covariance matrix obtained from the fit. To express the uncertainty in K_D_ itself, error propagation was applied, yielding

$$\sigma_{KD}=ln\left(10\right){\cdot K}_{D}{\cdot \sigma }_{\log_{10}\left(K_{D}\right)}$$

in line with established methods for uncertainty quantification in nonlinear regression [56].

### 4.3. Protein–Protein Docking

Docking between BvPgb 1.2 (PDB ID: 7ZOS) and human placental GAPDH (PDB ID: 1U8F) was performed using the HADDOCK2.4 web server. Input structures were cleaned by removing solvents and water and relabeling the heteroatoms of ligands. Docking was performed with higher allowance for restraint violations to increase conformational sampling of the putative interaction interface. Residues were chosen to not interfere with the known dimerization interface for BvPgb 1.2 (as can be seen in PDB ID: 7Z1U), with the same approach applied for GAPDH. For BvPgb 1.2, a total of 19 active residues (25, 28, 29, 64, 65, 68, 71, 72, 75, 76, 79, 83, 86, 91, 92, 93, 95, 96, 99) were selected further based on surface accessibility. GAPDH was assigned 11 active residues (25, 26, 29, 55, 57, 58, 59, 70, 71, 72, 73) on the O-chain. Passive residues were automatically defined within a 6.5 Å radius from the active sites, with buried residues filtered out using a burial cutoff of 15 Å^2^.

Docking was carried out with 10,000 rigid-body docking models, followed by 400 semi-flexible refinement models and 400 explicit solvent-refined models. The final refinement stage involved short molecular dynamics simulations in a shell of explicit water molecules. Clustering of the refined models was carried out using the FCC method with a 0.6 Å RMSD cutoff. The HADDOCK scoring function included contributions from vdW, electrostatic, and desolvation energies. The top-ranked cluster, selected based on the HADDOCK score, was analyzed for interface energy components, structural convergence, and BSA. Structures and input parameter files are available upon request.

### 4.4. Protein–DNA Docking

Docking between BvPgb 1.2 (PDB ID: 7ZOS) and B-form DNA (PDB ID: 1BNA) was performed using the HADDOCK2.4 web server, with similar cleaning and file preparation as in the protein–protein dock. For BvPgb 1.2, the same 19 residues as above were selected as active for similar reasons. For DNA, 11 nucleotides were selected as active (5, 6, 7, 8, 9, 10, 14, 15, 16, 21, and 23). Passive residues were similarly assigned as above. Docking was carried out similarly as above, with custom annealing schedule better suited for docking nucleic acids (e.g., amb_hot = 10, amb_cool1-3 = 10/50/50), electrostatics simulated with a constant permittivity (ε = 78.0), retention of groove-associated waters (dnap_water_tokeep = 0.75), and advanced water analysis. Clustering and analysis were performed as above. Structures and input parameter files are available upon request.

### 4.5. Sequence and Structural Characterization of Putative B. vulgaris GAPC

The amino acid sequence of *Arabidopsis thaliana* cytosolic GAPDH (GAPC1; UniProt accession P25858) [35] was used as a query in BLASTp (NCBI) searches against the *B. vulgaris* RefBeet-1.2.2 genome assembly [36]. The top-scoring alignment was selected based on low E-value and high sequence identity. The protein sequence was submitted to AlphaFold3 [37] for structure prediction. The resulting *B. vulgaris* GAPC model was structurally aligned with the GAPDH protein structure file used in the protein–protein dock earlier using the matchmaker tool in UCSF ChimeraX [38]. Alignment quality was assessed visually and by RMSD.

## 5. Conclusions

This study demonstrates that BvPgb 1.2 can directly interact with both GAPDH and DNA, with affinities strongly influenced by the conserved C86 residue. The dual-emission SpS measurements revealed biphasic DNA binding and a stable protein–protein interaction, findings that were corroborated by docking analyses showing electrostatically driven interfaces near the heme pocket. Importantly, these models suggest that ligand accessibility is preserved. Future studies should investigate the in vivo contexts where these interactions are most relevant and aim to elucidate whether GAPC is involved in the plastidic trafficking of Pgbs.

## Figures and Tables

**Table 1:** Dissociation constants (K_D_s) measured through SpS for BvPgb 1.2 rWT and the C86A mutant.

	K_D(GAPDH)_	K_D(DNA,open)_ ^1^	K_D(DNA,closed)_ ^1^
Protein	[nM]	[nM]	[pM]
rWT	260 ± 50	120 ± 50	100 ± 10
C86A	120 ± 40	50 ± 20	230 ± 60

^1^ The DNA molecule is the pET19b vector, and the K_D_s are subscripted as adopting the open/closed configuration of the hexacoordinated globin accounts for the biphasic behavior as seen elsewhere [27].

**Table 2:** Statistics of BvPgb 1.2–GAPDH clusters with negative Z-score from HADDOCK.

	Cluster 1	Cluster 2	Cluster 5
HADDOCK score	−136.7 ± 0.6	−107.8 ± 3.0	−105.5 ± 5.3
Cluster size	324	11	10
RMSD ^†^ (Å)	1.4 ± 0.8	15.4 ± 0.0	9.7 ± 0.1
van der Waals energy (kcal/mole)	−76.6 ± 4.2	−67.5 ± 3.9	−55.1 ± 1.5
Electrostatic energy (kcal/mole)	−349.8 ± 39.9	−236.3 ± 26.3	−312.6 ± 40.6
Desolvation energy (kcal/mole)	9.2 ± 4.9	5.6 ± 1.7	9.6 ± 2.8
Restraints violation energy (kcal/mole)	7.0 ± 3.0	12.6 ± 3.2	24.8 ± 12.4
Buried surface area (Å^2^)	2463.4 ± 146.5	2357.1 ± 93.0	1984.4 ± 104.3
Z-score	−2.0	−0.5	−0.3

^†^ From overall lowest-energy structure.

**Table 3:** Statistics of BvPgb 1.2–DNA clusters with negative Z-scores from HADDOCK.

	Cluster 3	Cluster 1	Cluster 4	Cluster 2
HADDOCK score	−119.1 ± 7.3	−108.5 ± 8.6	−94.6 ± 1.8	−93.7 ± 4.0
Cluster size	80	97	24	83
RMSD ^†^ (Å)	0.9 ± 0.6	4.2 ± 0.9	2.7 ± 0.2	13.6 ± 0.1
van der Waals energy (kcal/mole)	−69.0 ± 5.9	−65.5 ± 6.8	−48.1 ± 2.3	−61.0 ± 5.7
Electrostatic energy (kcal/mole)	−353.5 ± 23.2	−296.6 ± 35.0	−323.0 ± 18.6	−232.9 ± 46.9
Desolvation energy (kcal/mole)	16.0 ± 3.0	12.5 ± 1.4	16.9 ± 3.8	10.1 ± 3.1
Restraints violation energy (kcal/mole)	46.9 ± 26.5	38.4 ± 10.9	12.8 ± 15.7	37.9 ± 9.3
Buried surface area (Å^2^)	1701.4 ± 37.9	1632.2 ± 20.1	1497.4 ± 56.6	1424.3 ± 73.5
Z-score	−2.1	−1.4	−0.5	−0.4

^†^ From overall lowest-energy structure.

## Data Availability

Data supporting the findings of this study are available upon request from the corresponding author.

## Abbreviations

The following abbreviations are used in this manuscript:

BSA	Buried surface area
BvPgb 1.2	Class 1 phytoglobin from *Beta vulgaris*
C86A	Mutant of BvPgb 1.2 with cysteine-to-alanine substitution at position 86
Eair	Restraints energy (kcal/mole)
Edesolv	Desolvation energy (kcal/mole)
Eelec	Electrostatics energy (kcal/mole)
Evdw	vdW energy (kcal/mole)
FC	Ferrochelatase
FCC	Fraction of common contacts
GAPC	Cytosolic GAPDH in plants
GAPDH	Glyceraldehyde-3-phosphate dehydrogenase
Hb	Hemoglobin
i-RMSD	Interface RMSD (Å)
ITC	Isothermal titration calorimetry
K_D_	Dissociation constant (M)
LjPgb 3	Class 3 Phytoglobin from *Lotus japonicus*
Mb	Myoglobin
NAMPT	Nicotinamide phosphoribosyltransferase
NHS	N-Hydroxysuccinimide
PBS-T20	Phosphate-buffered saline supplemented with 0.05% Tween20
Pgb	Phytoglobin
rHb1	Class 1 phytoglobin from rice
RMSD	Root-mean-square deviation (Å)
ROS	Radical oxygen species
rWT	Recombinant wild type of BvPgb 1.2.
SpS	Spectral shift
vdW	van der Waal (kcal/mole)
